# The Application of Geometric Morphometrics to Explore Potential Impacts of Anthropocentric Selection on Animals' Ability to Communicate via the Face: The Domestic Cat as a Case Study

**DOI:** 10.3389/fvets.2020.606848

**Published:** 2020-12-21

**Authors:** Lauren R. Finka, Stelio P. L. Luna, Daniel S. Mills, Mark J. Farnworth

**Affiliations:** ^1^Animal, Rural and Environmental Sciences, Nottingham Trent University, Southwell, United Kingdom; ^2^School of Life Sciences, University of Lincoln, Lincoln, United Kingdom; ^3^School of Veterinary Medicine and Animal Science, São Paulo State University (Unesp), Botucatu, Brazil

**Keywords:** selective breeding, domestication, communication, signaling, facial expression, emotion, geometric morphometric analysis, *Felis silvestris*

## Abstract

During their domestication via artificial selection, humans have substantially modified the morphology and thus visual appearance of non-human animals. While research highlights the negative impact of these modifications on physical functioning, little is known about their impact on behavior and signaling, either toward humans or conspecifics. Changes in the appearance of the face, such as those associated with, but not limited to, facial expressions, form an important part of non-verbal communication. In companion animals, the face is one of their most visually diverse features (due to human-driven selection), which may impact the visual clarity of expressions and other forms of signaling. Using the domestic cat as our model, we applied a new analytical technique in order to understand the impact of breed variation on relative positioning of facial landmarks, chosen specifically for their association with the production of various facial movements, and the expression of affect. We then assessed the extent to which facial appearances known to be associated with a specific underlying state (i.e., pain, assessed via a validated, facial pain score), could be reliably detected in a morphologically diverse population. Substantial baseline variation in landmarks was identified at both the cephalic (e.g., brachycephalic, dolichocephalic, mesocephalic) as well as breed levels. While differences in facial pain scores could successfully differentiate between “pain” and “no pain” in the facial appearance of domestic shorthaired cats (DSH), these differences were no longer detectable when assessed within a larger more morphologically diverse population, after corrections for multiple testing were applied. There was also considerable overlap between pain scores in the DSH “pain” population and the neutral faces of other breeds. Additionally, for several paedomorphic breeds, their neutral face shapes produced scores indicative of greater pain, compared to most other breeds, including the DSH cats actually in pain. Our findings highlight the degree to which anthropocentric selection might disrupt the communicative content of animals' faces, in this case the domestic cat. These results also suggest a potential human preference for features extending beyond the infantile, to include negatively-valenced facial forms such as pain.

## Introduction

The domestication syndrome highlights the rapid, cross-species convergence of key phenotypic changes not usually seen in wild populations. Along with behavioral changes such as increased docility and tameness toward humans, domesticated animals may also exhibit common morphological changes associated with coat color, as well as ear and tail form, craniofacial morphology, and regional and overall brain size (1–3). Variation in human-driven selection for specific features has also led to much greater levels of intra-species behavioral and morphological diversity than may typically be present in their wild progenitors (4–6). In companion animals, such anthropogenic selection has contributed to the creation of breeds with exaggerated characteristics or various morphological “extremes,” affecting a range of features [e.g., the size and shape of their face and ears, their limbs, tails, and general body size and shape; (7–9)]. In more recent decades, some of this variation has been driven primarily by aesthetics, with human preferences prioritized over basic health or functioning (10, 11).

Visual behavioral expression relies on the production of changes to individuals' appearance, in order to convey important information. Because effective use of such information requires that the visual signals of the sender are clearly detectable and decodable by the recipient, signals must have elements of universality in their presentation. Companion animals are frequently exposed to various social pressures from humans, and may be expected to cohabit with conspecifics and/or other species, often under suboptimal conditions [e.g., (12–14)]. The value of effective communication in these contexts may be particularly high, yet it is possible that such animals might be at a disadvantage, given the radical morphological differences they display at a species level [e.g., (7, 9, 15)].

To date, research efforts have primarily focused on the effect of breed-based morphology on animals' physical functioning (16–18). In contrast, relatively little attention seems to have been paid to the way these morphological differences might impact their communicative abilities, with the focus being primarily on the domestic dog in this regard [e.g., (19–21)]. This is surprising, given the apparent contribution of breed-linked morphological differences to other aspects of dog's behavior, such as their visual acuity (22) and general cognition (23), as well as breed-linked differences in sociability (24), aggression (25), and maternal care (26) observed in various other domesticated species.

As in humans (27), changes in the visual appearance of the face, such as those caused by facial expressions, are likely to form a key aspect of visual communication in a range of animal species. Changes in expressions may be used to convey information about the producers' internal state or intentions (28–30), potentially serving important social and care solicitation functions. In recent decades however, the “default” visual appearance of the face has been notably altered via human selection, in a variety of companion animal breeds. For example, more paedomorphic or human infant-like features (i.e., a relatively large head and a round face, a high forehead and large, low-lying eyes) are now present in certain dog (31), cat (11), horse (8), and rabbit breeds (32). The “baby schema” hypothesis suggests that these features are particularly attractive to humans, triggering a nurturing response (33, 34). Indeed, in many cases, individuals with more paedomorphic characteristics can be perceived as cuter and preferred as pets (31, 35). Increased selection for this aesthetic as well as other morphological extremes, is potentially evidenced in the diversification of cephalic types present in all the aforementioned species (15, 32, 36, 37).

As well as the global shape of the face, human selection may also have altered specific muscles within it. For example, compared to wolves, domestic dogs have a more well-developed “inner eye brow raising muscle,” which is able to produce an expression that in humans is associated with sadness (38). The authors speculate that such expressions might indicate the dogs' communicative intent toward humans, and/or elicit a nurturing response from us (38). While dogs that produce this expression more frequently, may be more desirable to humans (31), the communicative value of this expression remains unknown, as does the degree to which this muscle is developed across breeds of dogs with varying facial morphology.

Although yet to be documented scientifically, it is likely that intra-species variations in facial morphology affect the dynamism of the face, the range of expressions and general shape changes possible, as well as their reliable visual detection. For example, the presence of permanent wrinkles on the face as a breed characteristic (such as in the British bulldog or Sphynx cat), may compromise the ability to detect the absence, presence, or intensity of facial expressions characterized by facial wrinkling. In horses, breed-linked variation in the features of eye wrinkles during neutral expressions has also been detected (39), potentially causing similar issues in this species. Other features of the face that display substantial breed variability, such as the ears, are also likely to be impacted. For example, the small, permanently folded ears in the Scottish fold cat have a very different appearance to the typical upright pinnae of other domestic cat breeds. The cartilage abnormalities causing this ear shape (40) are likely to limit the general motility of the ears, thus limiting the production of the various ear positions that are displayed at a species level [see (41, 42)].

To date, our scientific understanding of changes in the visual appearance of the face, such as those caused by facial expressions, and their relationship to animals' internal states, is relatively limited. However, some progress can be seen in relation to pain, with several key pain-linked changes in the appearance of the face identified and visual tools (e.g., facial grimace scales) created to assist in pain detection (29, 43, 44). These scales broadly rely on the identification of differences in the shape of various features within the face (corresponding to intensities in pain), which are identified relative to a specific baseline or neutral exemplar. However, the clinical application of this approach may be problematic in populations with diverse facial morphology (i.e., certain companion species), because the appearance of neutral faces in some breeds may resemble those of others following emotional change and thus, a species level baseline may be of limited practical use. This issue is potentially resolvable if individual baselines are available, although this requires their prior (reliable) collection and availability at the time at which an assessment takes place. Furthermore, the potential variability in the range of movement or functionality of the face across individuals with different facial morphologies, is likely to affect the relative apex of various facial movements. This may limit observer's ability to gauge the intensity of pain experienced, based on the degree of facial shape change observed in a specific animal, even when individual baselines are available.

Given the importance of the face in visual communication and expression, and the extent to which it has been altered for non-functional purposes in domesticated animals, further investigation is imperative. The recent, novel application of a geometric morphometric approach to the study of facial expressions in animals [e.g., (45)], provides a useful tool in this regard. Traditionally, morphometric measurements have involved the placing of points onto images (often of skeletal remains), with shape variations quantified based on a series of linear distances. More recent, landmark-based geometric approaches however, better facilitate holistic quantification and visualization of shape and its variation. In Finka et al. (45), this latter approach was used to measure facial shape changes associated with the expression of pain in domestic cats. A series of 48 facial landmarks (represented as 96, x-y coordinates) were placed on the external facial features of images taken from living individuals. These points were selected specifically due to their association with the repertoire of facial expressions displayed in this species (41, 42). Landmarks were located relative to cat-specific underlying facial musculature, as well as locations associated with changes in facial shape, caused by contractions of various muscles [sensu catFACS; (41, 42)]. Facial landmarks were annotated onto images manually and demonstrated very good inter-annotator reliability (45). This method was able to identify, and visually quantify, within-individual facial shape changes associated with the expression of pain, across varying intensities; the facial pain scores generated from configurations of landmarks also showed good convergent validity with another well-validated measure of pain assessment in cats (46).

This type of facial analysis therefore presents a novel way to investigate how the shape of the face changes as a consequence of landmark positions which are linked to facial musculature and the expression of internal states. By investigating the relative “baseline” locations of these landmarks in the neutral faces of static images of individuals within visually diverse populations, it is possible to investigate how affect-linked changes in facial shapes may cease to be distinguishable from the facial shape differences present as a result of morphological variation. In effect, this method can be used to investigate whether differences in facial morphology might serve to disrupt the expressive content and thus communicative value of the face, at a population level.

Domestic cats exhibit a diverse range of breed types and morphological characteristics. Driven primarily by aesthetics, morphological diversification from wild type has occurred relatively rapidly, with the majority of the currently recognized cat breeds established within the last century see (47, 48). Amongst these breeds, a broad range of cephalic shapes are evident. These range from brachycephalic; a more rounded skull shape and reduction in length of the face and braincase, such as in the Persian, to dolichocephalic; a comparatively decreased skull width but increased facial length, such as in the Siamese (15). At a species level, the domestic cat also displays a diverse range of facial expressions, the most commonly occurring of which have been systematically documented at an anatomical level. These are listed as a series of Action Units and linked to specific facial muscles (41, 42). The production of such facial Action Units, and other changes in facial shape, have been associated with affective states such as fear, frustration and relaxed engagement (49), as well as acute pain (50) and its intensity (45, 51). Such findings demonstrate that changes in cats' facial appearance can contain useful information relative to their internal state, and are likely of communicative importance.

Given the newly developed application of geometric morphometrics to the study of facial expressions in this species, the domestic cat provides a useful model to explore the potential impacts of anthropocentric selection on the expressive value of the face, at a species level. Our aims were therefore to understand how breed-based morphology impacts on the relative position, and thus appearance, of facial landmarks associated with the production of expressions in cats (study 1). We then assessed the extent to which facial appearances associated with an affective state (i.e., pain) in domestic short haired cats could be reliably detected within a population including these more diverse facial morphologies (study 2).

### Ethical Statement

Part of the dataset used for this study was collected previously for the purposes of validating a composite pain scale in domestic cats (46). Its use was approved by the Institutional Animal Research Ethical Committee of the FMVZ-UNESP-Botucatu under the protocol number of 20/2008. The use of this dataset and the generation of the data were approved by the delegated authority of Nottingham Trent University, Research Ethics Committee. All experiments were performed in accordance with relevant guidelines and regulations.

## Study 1 Methods

### Image Selection and Data Extraction

Unique facial images of common cat breeds (*n* = 19) were sourced by author LF from the Oxford Pet dataset (52) and Google images (see Supplementary Data 1 for full list of breeds). A total of 1,888 images across the breeds were manually annotated by LF, using the 48 x-y facial landmark model developed by Finka et al. (45). Annotations comprised a mixture of type I landmarks, placed relative to muscle insertion points, which move during muscle contraction and type ii landmarks, placed relative to the shape changes caused by various groups of muscle contractions, associated with cat Facial Action Units [see catFACS; (41)]. Specific details of landmark placements, their relevance to facial musculature and action units [as documented in Finka et al. (45)] are included in Supplementary Data 2.

Selection criteria for the inclusion/exclusion of images within our analysis were as follows. Each image had to be of a unique adult individual suitably representative of their labeled breed and displaying a “neutral” facial expression, with images taken in a seemingly neutral context i.e., at home rather than at a veterinary clinic or shelter, not being handled by humans, or partaking in any social interactions with either humans or other animals. The cat's appearance was studied to ensure it reflected the descriptors and visual examples of the breed standards as provided by The Cat Fanciers' Association (53), and The International Cat Association (54). Search terms used to source images were limited to the breed names as indicated in Supplementary Data 1. Domestic Short Haired cats were identified as those that had mesocephalic type features (see further) and did not display any distinct visual features that could enable then to be classified as any other recognized breed type. Knowledge of the cat Facial Action Coding System and the reference manual was used by LF (a certified reliable catFACS coder) to determine neutrality of expressions within the images [see (41)], with the aim to discount any cats displaying various muscle contractions or other expressions associated with affective states such as pain, fear or frustration [e.g., (45, 49–51)]. As these images were from unknown populations, no information relating to their geographical location, age, sex, neuter or health status was available, although images of kittens, cats with proportionately large jowls (as seen in some adult unneutered males) and cats which looked physically ill or in distress or were not included. The amount of space within a picture taken up by the cats' face varied between images, although was not considered an issue given the subsequent Procrustes procedure performed post annotation (see further). However, only images with a sufficient resolution to facilitate practical location of all 48 landmarks were included.

To enable the practical sourcing of sufficient unique images, those with a degree of lateralised pose (i.e., the cat facing toward the camera lens but not in a perfect frontal position) were also included, as long as all 48 landmark features could be clearly annotated. This was considered acceptable given that the graphical images (i.e., lollipop graphs representing the direction and magnitude of landmark displacement associated with each PC – see further) produced with this method made it possible to visually identify shape variation likely to be caused by pose than by morphological differences.

Each breed was also assigned to one of the three broad cephalic categories (e.g., mesocephalic, brachycephalic and dolichocephalic), based on visual inspection of general face shapes that were typical for the majority of images (at least 90%) within that breed. A breed was categorized as brachycephalic where the majority of sourced images depicted cats with comparatively shortened muzzle and cranial lengths and nose to eye distances, as dolichocephalic where the majority of images depicted cats with comparatively elongated muzzle and cranial lengths and longer nose to eye distances and as mesocephalic where the majority of images were of cats with comparatively proportioned features with no obvious shortening or lengthening [see (55)]. While this method of categorization likely meant than certain breeds in each category would be more “extreme” representatives of their cephalic type than others, variability within breeds was controlled for as follows. Any images depicting cats whose features did not clearly align to the cephalic category assigned to their breed were treated as outliers and not included in the final set of images. Additionally, breeds such as the Siamese were not included, due to the apparent diversity in their facial morphology across “modern” and “traditional” types, ranging from relatively mesocephalic to more dolichocephalic. Images were subsequently checked by author MF to ensure agreement with both the breed and cephalic categories to which images had been assigned. Subsequent contributions from each breed ranged between 89 and 107 unique examples, depending on practical availability of suitable images. Images were digitized using ImageJ: version 1.49v (56) and landmark configurations extracted in order to create a geometric representation of the cat's face in the form of 48 paired x-y coordinates.

### Identifying Key Sources of Facial Shape Variation Within the Population

Analyses followed a similar method to that of Finka et al. (45). The extracted landmark configurations were subjected to Procrustes superimposition to remove scaling, rotation and translation effects, using MorphoJ (57). A Principal Component Analysis (PCA) was then performed on the Procrustes coordinates (58). Scree plots were assessed and meaningful PC components (based upon their proportion of variation explained) were retained for visualization and further analysis. Relative changes in facial landmark locations represented by each of these PCs were visualized via lollipop graphs. Each lollipop graph is generated directly from the relative loadings of each x-y coordinate within a given principal component. The circular nodes of the lollipop graphs represent the average position of facial landmarks, with the lines protruding from each landmark highlighting the direction and magnitude of relative shape change. Higher PC scores (thus greater landmark displacement in a given direction) are reflected by a greater distance along the line from the circular nodes, with lower PC scores reflecting less distance from the nodes. This method was also used to detect where PCs were likely to represent variation relating to lateralised pose (e.g., indicated by obvious lateral differences in node length and direction).

### Identifying Variability in Shape Morphology at the Cephalic and Breed Level

The retained principal components were then used to generate scores for each image, based on the weighted loading of each coordinate within a given component (see Supplementary Data 3). Shape variations across cephalic types were assessed for each PC via Kruskal-Wallis and subsequent Mann-Whitney U tests for *post hoc* pairwise comparisons. To give a simple overview of general variability at the breed level, shape differences were first assessed collectively for all retained PCs via one-way non-parametric permutational MANOVAs (NPERMANOVAs) with *post hoc* tests. Relative shape variation was then assessed in more detail, analyzing each PC separately, using Kruskal-Wallis and subsequent Mann-Whitney U tests as above. In all cases, outputs were subjected to Bonferroni corrections. Tests were performed using Past, version 3.10 (59). Wireframes based on the average landmark configurations for each cephalic type (mesocephalic, brachycephalic and dolichocephalic) and also for each breed, were produced using MorphoJ (57). These enabled visual comparison of average global differences in baseline facial landmark positions, and their variation, across the different populations.

## Study 1 Results

### Key Sources of Shape Variation Within the Population (*n* = 1,888)

The first four principal components collectively explained 79% of the variation within the population (PC1 32%, PC2 27%, PC3 13% and PC4 7%), with additional components contributing little extra variation (i.e., 4% or less). Lateralised differences in landmark displacements were generally absent from PCs 1, 3 and 4 and prominent within PC2. PC2 was therefore interpreted as primarily relating to pose effects, rather than differences in the baseline locations of landmarks caused by variation in underlying facial morphology. Landmark displacements associated with PC2 were therefore not considered in subsequent analyses.

**Figure 1:**
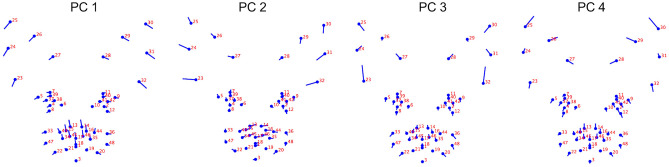
Geometric representation of the face based on average landmarks from 1,888 unique images of cats across the 19 different breeds. Lollipop graphs depict the relative facial shape changes associated with higher PC scores for PCs 1-4. The lines protruding from each landmark indicate direction and magnitude of movement, with higher PC scores reflecting a greater distance along the line from the circular nodes. Images produced using MorphoJ, Version 1.06d (57).

For PC1, co-ordinates relating to the ears (landmarks 23–32), eyes (landmarks 4, 5, 7, 8, 37–39 and 9, 11, 12, 40–42) and nose (landmarks 13–18, 35–36, 44–46) loaded prominently, with an increase in component score indicating greater displacement in a lateral and ventral direction for all points positioned along the pinnae, a greater lateral displacement of the medial and lateral points around the eyes, and a greater displacement of the points located on the nose in a dorsal direction toward the eyes (see Figure 1).

For PC3, co-ordinates relating to the ears (landmarks 23–32) also loaded prominently, however in this case, the direction of their displacement indicated a proximal difference in the landmarks at the top of the pinnae (landmarks 25 and 30), a medial displacement in the landmarks at the mid points of the pinnae (landmarks 24, 26, 29, 31), a dorsal displacement in the landmarks at the outer base of the pinnae (landmarks 23 and 32), and a dorsolateral displacement in those positioned at the medial pinnae bases (landmarks 27, 28). To a lesser degree, co-ordinates around the lateral edges of the whisker pads (landmarks 20, 47, 33, and 20, 48, 36) indicated displacement in a ventrolateral direction (see Figure 1).

For PC4, co-ordinates for the ears (landmarks 24–31), eyes (landmarks 4–12, 37–42), nose (landmarks 13–18, 34, 35, 43–46), and cheeks (landmarks 20, 22, 33, 36, 47, 48) all loaded prominently. For the ears, an increase in PC4 score indicated a greater displacement of the dorsal and mid points of the pinnae in a dorsomedial direction, and displacement of the medial pinnae bases in a ventromedial direction. For the eyes, displacement occurred in a ventrolateral direction. For the nose, displacement was predominantly in a dorsal direction, and for the cheeks, in a ventral or ventrolateral direction (see Figure 1).

### Shape Variation Across Cephalic Types (*n* = 1,888)

#### Variation in PC1 Scores

**Figure 2:**
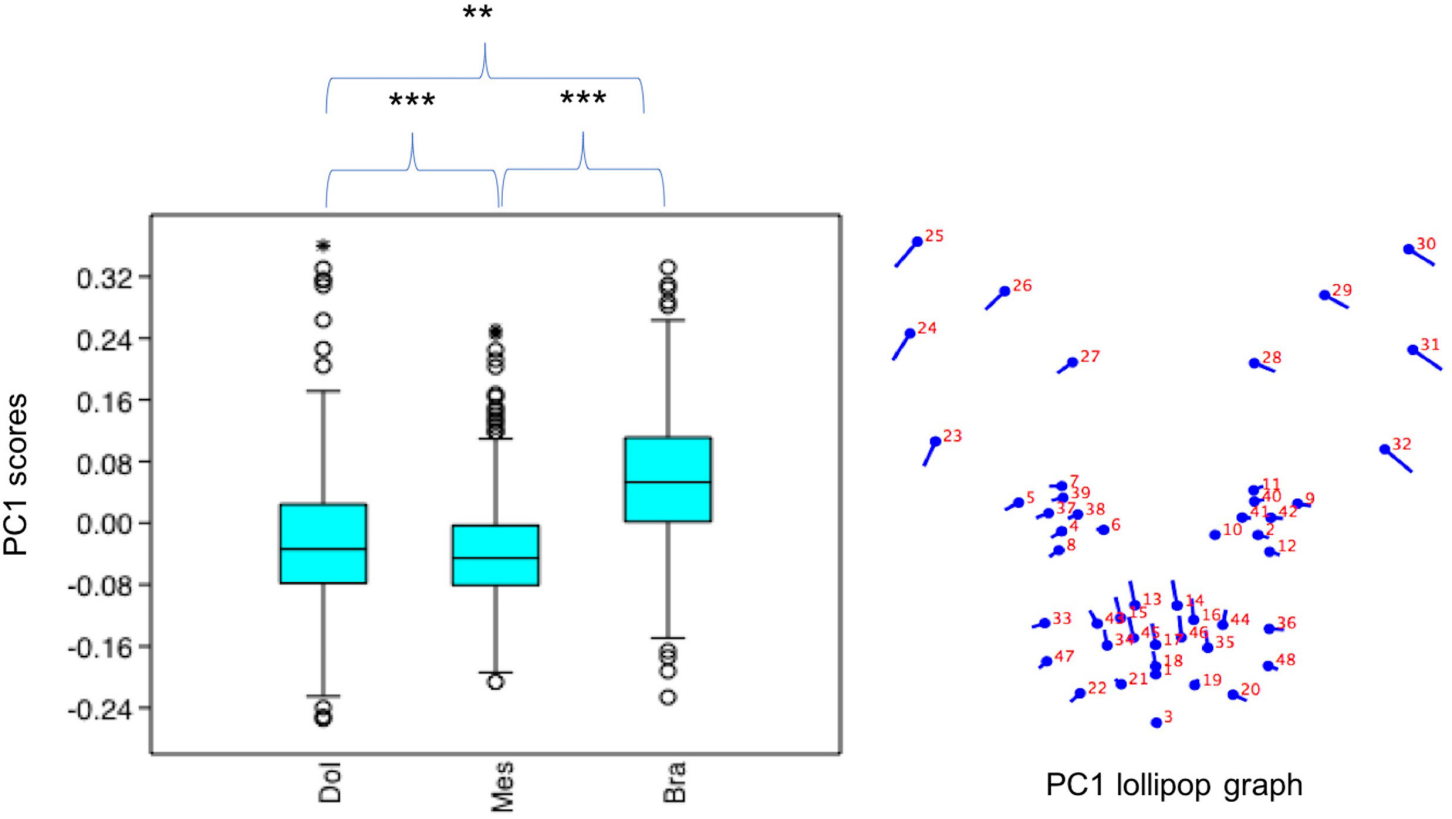
Boxplot with minimum, first quartile, median, third quartile, maximum values and outliers for PC1 scores for each of the cephalic types; Dol, dolichocephalic; Bra, brachycephalic; Mes, mesocephalic; *n* = 1888. **p < 0.01, ***p < 0.001. Relative landmark variation for PC1 represented via lollipop graph. Lines protruding from each landmark indicate direction and magnitude of movement, with higher PC1 scores reflecting a greater distance along the line from the circular nodes. Images produced using MorphoJ, Version 1.06d (57).

PC1 scores varied significantly across cephalic face types (*x*^2^ = 486.8, *p* = 1.925e-106). Pairwise comparisons indicated PC1 scores for brachycephalic faces were significantly higher compared to both mesocephalic (*u* = 8.566e04, *p* = 3.76e-97) and dolichocephalic (*u* = 7.848e04, *p* = 1.373e-55) types, and PC1 scores significantly higher for dolichocephalic, compared to mesocephalic faces (*u* = 1.559e05, *p* = 0.007473), see Figure 2.

#### Variation in PC3 Scores

**Figure 3:**
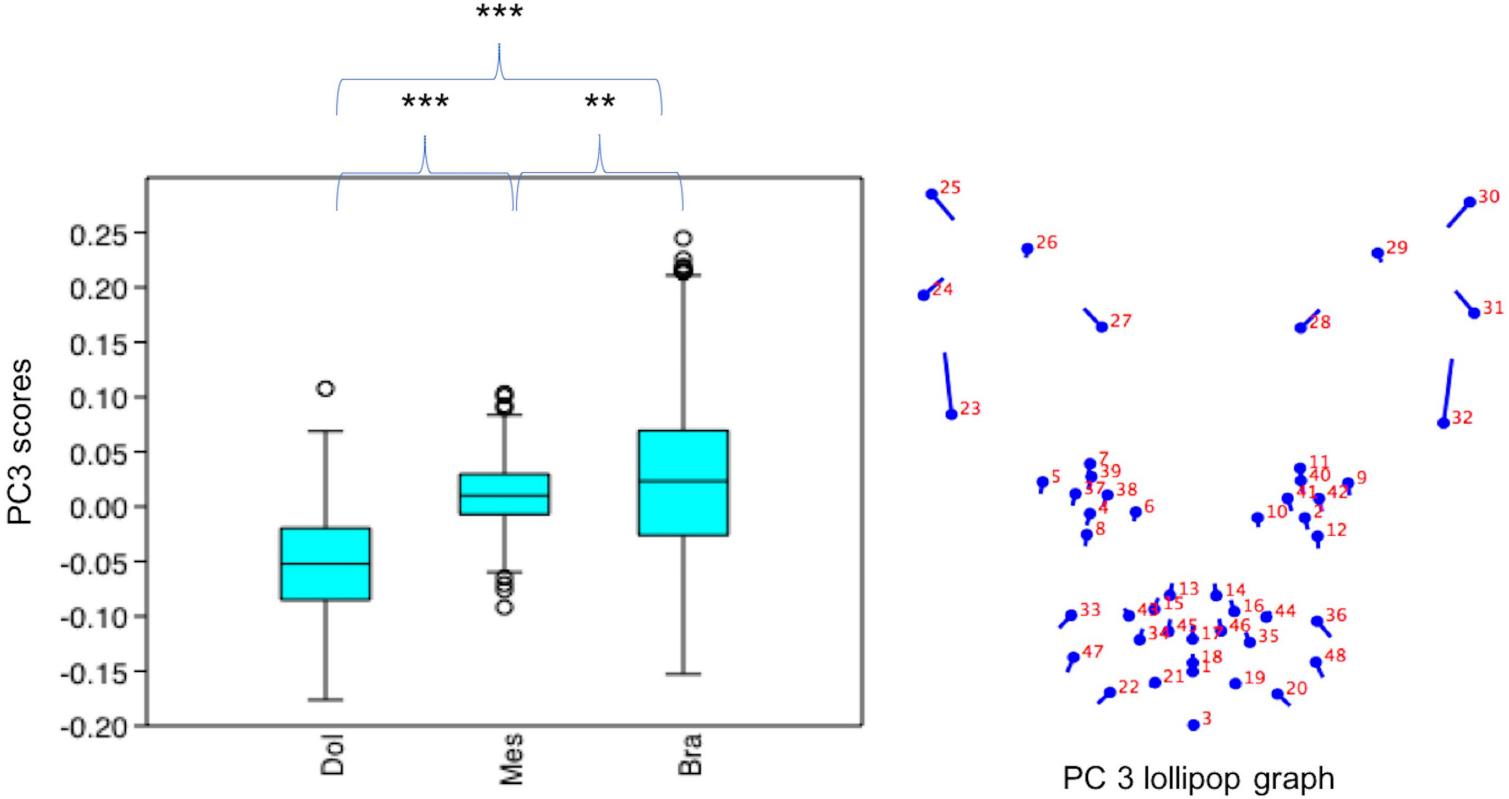
Boxplot with minimum, first quartile, median, third quartile, maximum values and outliers for PC3 scores for each of the cephalic types; Dol, dolichocephalic; Bra, brachycephalic; Mes, mesocephalic; *n* = 1,888. **p < 0.01, ***p < 0.001. Relative landmark variation for PC3 represented via lollipop graph. Lines protruding from each landmark indicate direction and magnitude of movement, with higher PC3 scores reflecting a greater distance along the line from the circular nodes. Images produced using MorphoJ, Version 1.06d (57).

PC3 scores varied significantly across cephalic face types (*x*^2^ = 524.3, *p* = 1.39e-114). Pairwise comparisons indicated PC3 scores for brachycephalic faces were significantly higher than for both mesocephalic (*u* = 2.167e05, *p* = 0.001054) and dolichocephalic faces (*u* = 6.358e04, *p* = 9.703e-75), and PC3 scores significantly higher for mesocephalic faces compared to dolichocephalic faces (*u* = 4.228e04, *p* = 1021e-109), see Figure 3.

#### Variation in PC4 Scores

**Figure 4:**
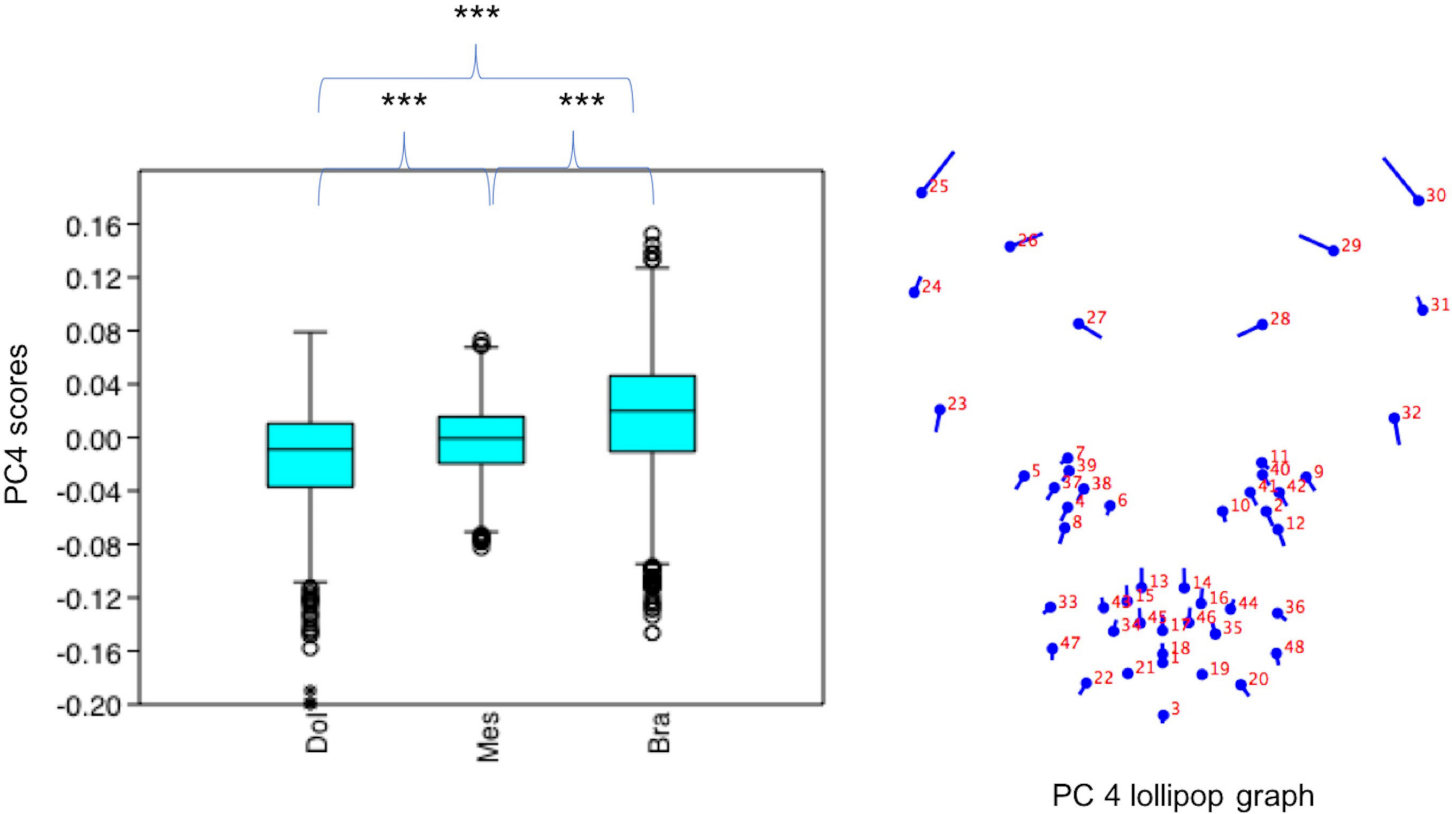
Boxplot with minimum, first quartile, median, third quartile, maximum values and outliers for PC4 scores for each of the cephalic types; Dol, dolichocephalic; Bra, brachycephalic; Mes, mesocephalic; *n* = 1,888. ***p < 0.001. Relative landmark variation for PC4 represented via lollipop graph. Lines protruding from each landmark indicate direction and magnitude of movement, with higher PC4 scores reflecting a greater distance along the line from the circular nodes. Images produced using MorphoJ, Version 1.06d (57).

PC4 scores varied significantly across cephalic face types (*x*^2^ = 156.2, *p* = 1.224e-34). Pairwise comparisons indicated PC4 scores for brachycephalic faces were significantly higher compared to both mesocephalic (*u* = 1.749e05, *p* = 2.38e-11) and dolichocephalic faces (*u* = 1.05e05, *p* = 1.339e-28), and PC4 scores significantly higher for mesocephalic compared to dolichocephalic faces (*u* = 1.427e05, *p* = 4.403e-07), see Figure 4.

#### General Visual Differences Across Average Face Shapes for Each Cephalic Type (see Also Figure 5)

Inspection of the wireframes, based on average landmark configurations for each cephalic type, indicated the following key visual differences in shape variation:

- In the brachycephalic face, a smaller distance between the baseline positions of landmarks for each pinna, but a greater distance between pinnae. Landmarks at the lateral edges of the eyes positioned more toward the horizontal, and the nose landmarks closer toward those of the eyes. Landmarks on the lateral edges of the cheek and mouth areas positioned further away from those of the nose and eyes.
- In the dolichocephalic face, baseline positioning of the same landmarks indicated relative differences in the opposite directions from those of the brachycephalic face.
- In the mesocephalic face, relative landmark positions appeared mid-way between the two other face types.

**Figure 5:**
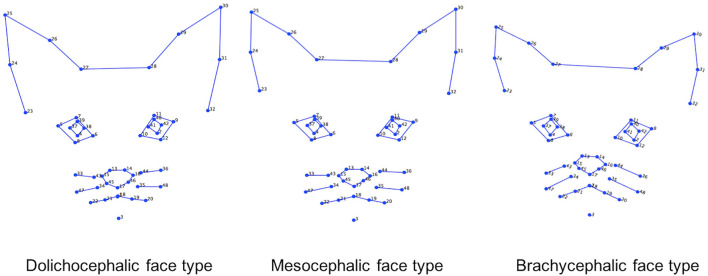
Geometric wireframes created for each of the three main cephalic face types. Wireframes are based on average landmark positions for each cephalic type (*n* = 1,888). Wireframes produced using MorphoJ, Version 1.06d (57).

### Shape Variation Across Breeds (*n* = 1,888)

#### Summary of Variation in Collective PC Scores Across Breeds (see Supplementary Data 4 for Full Results of NPERMANOVAs)

Breeds were significantly different from one another, based on their collective PC1, 3 and 4 scores (*f* = 109.9, *p* = 0.001). Pairwise comparisons across breeds indicated none of the mesocephalic breeds varied significantly from each other (*p* > 0.05), although they were all significantly different from all of the breeds within the brachycephalic group, as well as most of the breeds within the dolichocephalic group (*p* < 0.05). The exception to this was for four of the following mesocephalic breeds; Domestic short-hair, Main coon, Norwegian Forest cat and Russian Blue, which showed overlap with either one or both of the dolichocephalic breeds, Bengal and Egyptian Mau (*p* > 0.05).

Breeds within the dolichocephalic groups were significantly different to each other, as well as to mesocephalic and brachycephalic breed types (all *p* < 0.05), with the exception of the Bengal and Egyptian Mau (which overlapped with several of the mesocephalic breeds) and Sphynx (which overlapped with Devon Rex cats from the brachycephalic group).

All breeds within the brachycephalic group were significantly different from each other and from breeds from the other face types (*p* < 0.05), with the exception of the overlap between Devon rex and Sphynx cats (*p* > 0.05).

#### Summary of Variation in Individual PC Scores Across Breeds (see Supplementary Data 5 for Full Results of Mann-Whitney *U*-Tests)

**Figure 6:**
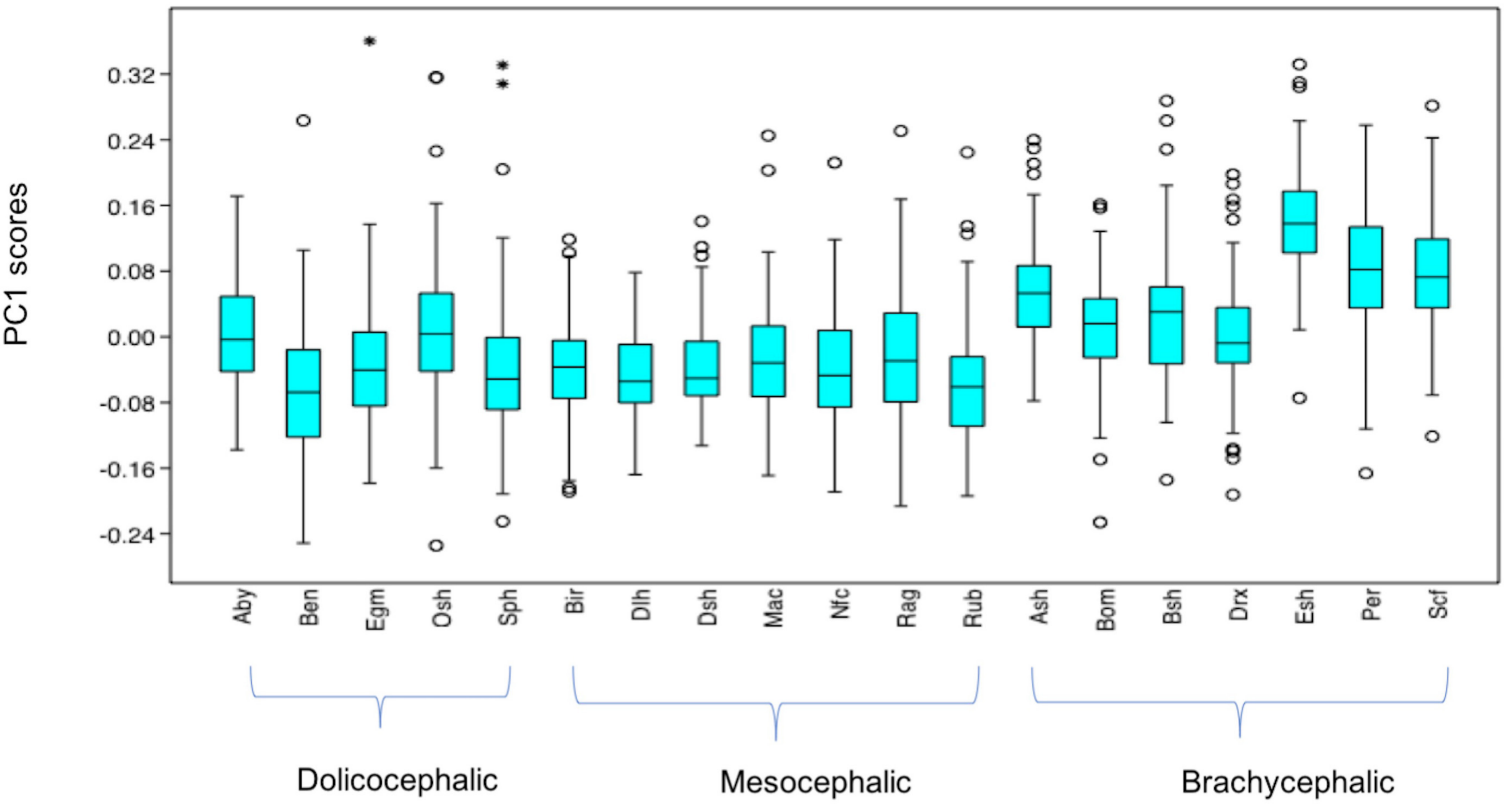
Boxplot with minimum, first quartile, median, third quartile, maximum values and outliers (* denoting more extreme outliers) for PC1 scores for each breed, *n* = 1,888. Abbreviations for breeds within each cephalic type as follows. Dolichocephalic: Aby, Abyssinian; Ben, Bengal; Egm, Egyptian Mau; Osh, Oriental Shorthair; Sph, Sphynx. Mesocephalic: Bir, Birman; Dlh, Domestic Longhair; Dsh, Domestic shorthair; Mac, Main Coon; Nfc, Norwegian Forest cat; Ragdoll, Russian Blue. Brachycephalic: Ash, American Short hair; Bom, Bombay; Drx, Devon Rex; Esh, Exotic Short hair; Per, Persian; Scf, Scottish fold. For full results of Mann Whitney *U*-tests see Supplementary Data 5. For shape changes associated with higher PC1 scores, see Figure 1. *Indicates significant individual outliers from the population.

**Figure 7:**
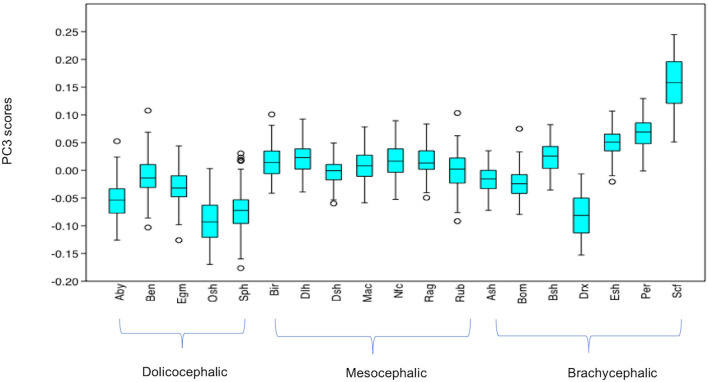
Boxplot with minimum, first quartile, median, third quartile and maximum values and outliers for PC3 scores for each breed, *n* = 1,888. Abbreviations for breeds within each cephalic type as follows. Dolichocephalic: Aby, Abyssinian; Ben, Bengal; Egm, Egyptian Mau; Osh, Oriental Shorthair; Sph, Sphynx. Mesocephalic: Bir, Birman; Dlh, Domestic Longhair; Dsh, Domestic shorthair; Mac, Main Coon; Nfc, Norwegian Forest cat; Ragdoll, Russian Blue. Brachycephalic: Ash, American Short hair; Bom, Bombay; Drx, Devon Rex; Esh, Exotic Short hair; Per, Persian; Scf, Scottish fold. For full results of Man Whitney *U*-tests see Supplementary Data 5. For shape changes associated with higher PC3 scores, see Figure 1.

**Figure 8:**
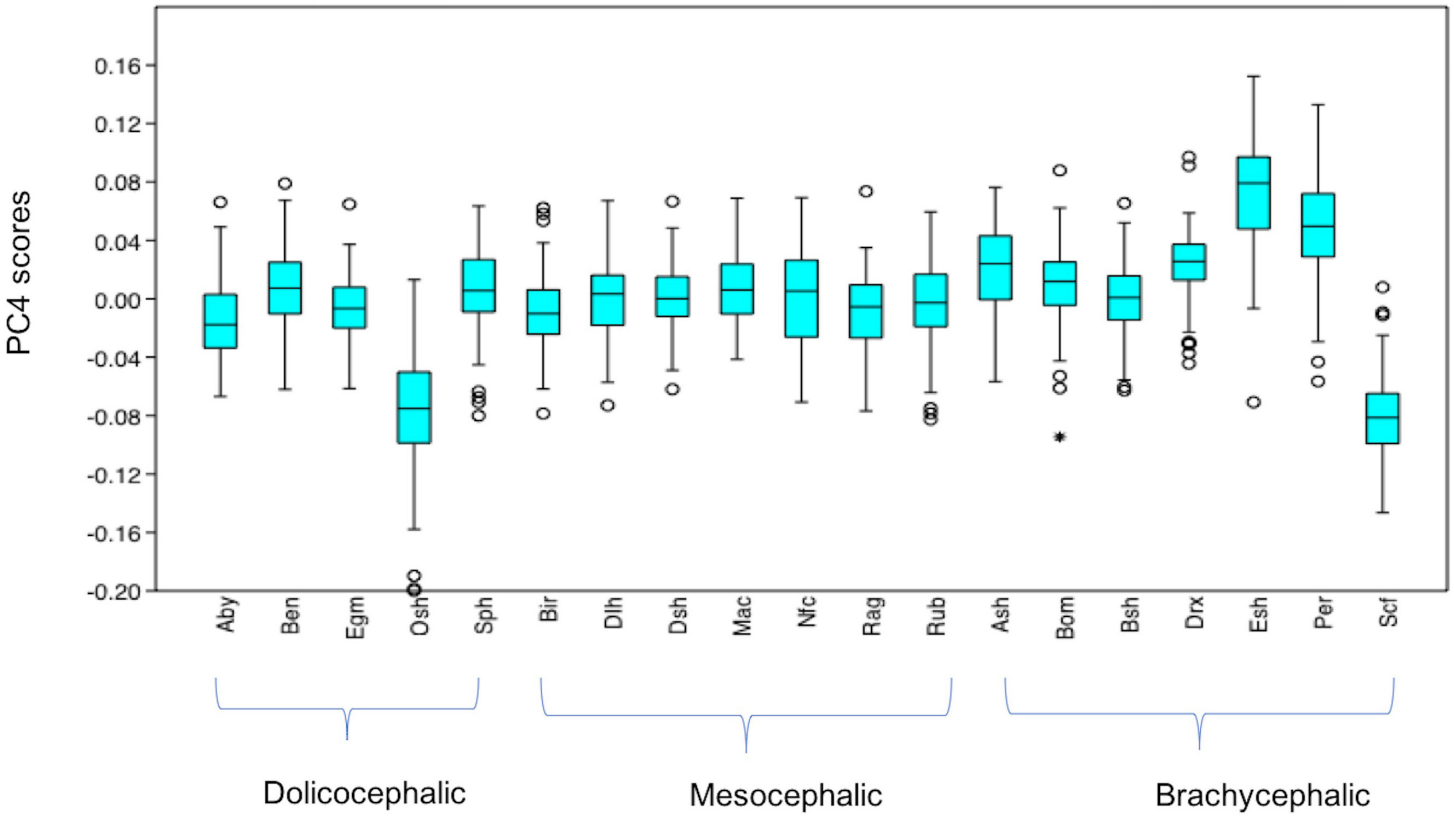
Boxplot with minimum, first quartile, median, third quartile, maximum values and outliers (* denoting more extreme outliers) for PC4 scores for each breed, *n* = 1,888. Abbreviations for breeds within each cephalic type as follows. Dolichocephalic: Aby, Abyssinian; Ben, Bengal; Egm, Egyptian Mau; Osh, Oriental Shorthair; Sph, Sphynx. Mesocephalic: Bir, Birman; Dlh, Domestic Longhair; Dsh, Domestic shorthair; Mac, Main Coon; Nfc, Norwegian Forest cat; Ragdoll, Russian Blue. Brachycephalic: Ash, American Short hair; Bom, Bombay; Drx, Devon Rex; Esh, Exotic Short hair; Per, Persian; Scf, Scottish fold. For full results of Mann Whitney *U*-tests see Supplementary Data 5. For shape changes associated with higher PC4 scores, see Figure 1.

Individually, each of the PCs also varied significantly amongst breeds (PC1: *x*^2^ = 698.8, *p* = 1.022e-136, PC3: *x*^2^ = 1,324, *p* = 3.68e-270, PC 4: *x*^2^ = 869, *p* = 6.543e-173). Pairwise comparisons between breeds indicated various significant differences in face shapes, both within the same, as well as across different cephalic face types, although some breeds were significantly different to each other on some PCs but not others. For example, PC scores for Oriental short-haired (dolichocephalic) cats were significantly different from Devon Rex (brachycephalic) cats for PC4 (*u* = 164, *p* = 1.952e-29), but not PC1 (*u* = 4,450, *p* = 1) or PC3 (*u* = 4,045, *p* = 1). In another example, PC scores for Abyssinian (dolichocephalic) cats were significantly different from British Short haired (brachycephalic) cats for PC3 (*u* = 349, *p* = 1.7e-27) and PC4 (*u* = 3,310, *p* = 0.009372), but not PC1 (*u* = 4062, *p* = 1). As with the combined PC scores, brachycephalic breed types were generally the most consistently different to other breeds, both within and across their face type, and across each PC. By contrast, mesocephalic breeds showed a much greater degree of overlap with other breeds. Trends for dolichocephalic breeds were somewhere in between those for other face types. In general, within breed variability in PC scores was greater for PC1, compared to PC3 and PC4, with several of the dolichocephalic breeds demonstrating some of the greatest degree of variability in PC1 scores (see Figures 6–8).

#### General Visual Differences Across Average Face Shapes for Each Breed (see Also Figure 9)

Inspection of the wireframes, based on average landmark configurations for each breed type, indicated the following key visual differences in shape variation. These occurred across each region of the face and associated landmarks, including:

- Lateral and dorsal variation in the distance between the landmarks on each pinna, and lateral variation in the distance between the pinnae.
- Variation in the distance between landmarks located around the cheeks and mouth, relative to landmarks around the nose and eyes.
- Lateral and dorsal variation in the distance between the landmarks on each eye, and their relative distance from landmarks of the ears and nose.

**Figure 9:**
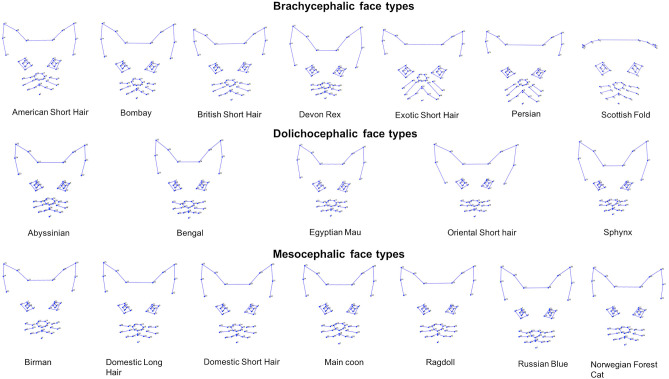
Geometric wireframes created for each of the 19 breeds. Wireframes are based on average landmark positions of each breed (*n* = 1,888). Wireframes produced using MorphoJ, Version 1.06d (57).

## Study 2 Methods

### Image Selection, and Data Extraction

Fifty facial images were obtained from a population of mixed breed (i.e., Domestic short-haired type) cats (2.8 ± 0.5 kg; 14.1 ± 5.2 months of age) undergoing ovariohysterectomy as described in Brondani et al. (46). These data were originally generated during the development of a previous postoperative composite pain detection scale [UNESP-Botucatu MCPS; Brondani et al. (46) and were then subsequently used to develop a geometric morphometric method to assess facial expressions in DSH cats (45)]. In Finka et al. (45) facial images were extracted from cats (*n* = 25) that were filmed across four conditions; before surgery (T1), 1 h post-surgery, before rescue analgesia (T2), post-rescue analgesia (T3), and 24 h post-surgery (T4). Cats were anesthetized prior to surgery (between T1 and T2) with propofol^a^ IV (8 mg/kg), fentanyl^b^ (0.002 mg/kg) IV and isoflurane^c^ in 100% oxygen. Postoperative analgesics were administered ~1 h post-surgery (Morphine^d^ (0.2 mg/kg) IM, ketoprofen^e^ (2 mg/kg) SC, and dipyrone^f^ (25 mg/kg) IV). Significant differences in cat face shapes across these conditions, relative to pain intensity, were identified within a single “pain” principal component (45). Face shape changes associated with a greater presence of pain included a more lateral and ventral positioning of the ears, a more dorsal positioning of the cheeks and mouth, a reduced distance between the cheeks, mouth and eyes, a narrowed eye aperture, lateral differences of the outer pinnae edges, and a left lateral position of the nose.

For the purposes of this study, we used images from only T2 (*n* = 25) and T3 (*n* = 25) because these were the two conditions where the most distinct differences in facial shapes were identified and as such were considered to represent the prototypical differences between the presence and absence of pain (45). Although averaged contributions from multiple images per cat are likely to provide a more reliable representation of a cat's face within a given state (49), for the purposes of this study, we used a single image from each cat from T2 and T3, so that these data could be directly compared to the single images from cats across the different breed groups. Images were randomly selected from the total number of images that were extracted for each cat from video footage collected during T2 (between 30 min and 1 h after the end of surgery, and prior to administration of additional analgesics) and T3 (~4 h after postoperative analgesia). Images were annotated using the same methods as in study 1, with the same criteria used to confirm DSH status as well as image selection [i.e., a degree of lateralised pose was acceptable, the cat was not being physically handled at the time of image extraction, see (45)].

A “pain PC score” was then generated for T2 (*n* = 25) and T3 (*n* = 25) images, based on the weighted loading of each coordinate on the pain PC [(45), see also Supplementary Data 10]. To confirm whether differences in pain PC scores were still evident between T2 and T3 using scores from single contributions rather than averaged values from multiple images [as in Finka et al. (45)], a Wilcoxon signed rank test was performed on the T2 and T3 pain PC scores.

### Identifying Variability in Pain PC Scores Across Cephalic Face Types and Breeds

Twenty-five images were randomly selected from each of the breed groups from study 1 (the neutral faces of cats across the 19 different breeds, *n* = 475). Pain PC scores for each image were then generated, using the same method as described above. Potential differences in pain PC scores in this population were explored, grouping the data based on cephalic face type. The pain PC scores for DSH cats “in pain” (T2) (*n* = 25) and “not in pain” (T3) (*n* = 25), were then added to this dataset and data were then grouped at the breed level, so that differences could be compared between the different breeds and also T2 and T3. In both cases, Kruskal-Wallis and subsequent Mann-Whitney *U*-tests with Bonferroni correction for *post hoc* pairwise comparisons were applied. All tests were performed using Past, version 3.10 (59).

## Study 2 Results

### Differences in Pain PC Scores Between T2 (“Pain”) and T3 (“No Pain”) in DSH Cats (*n* = 50)

In line with previous findings (45), differences in pain PC scores were still detectable using single images of DSH cats rather than averaged values from multiple images. Pain PC scores were significantly lower (indicating greater presence of pain) in the “pain” (T2) compared to the “no pain” (T3) DSH population (*z* = 268, *p* = 0.00453). However, when multiple comparisons were then made across T2, T3 and examples from the other breeds (those from study 1, see further in results) the strength of this relationship dropped (i.e., T2 scores were only significantly lower than T3 (*u* = 201, *p* = 0.03126) prior to Bonferroni correction). A similar pattern also emerged for the differences between PC scores for DSH cats in pain (T2) and the separate population of DSH cats with neutral expressions (those derived from study 1); T2 scores were significantly lower (*u* = 286, *p* = 0.0003586), but again only prior to Bonferroni corrections.

### Differences in Pain PC Scores Across Cephalic Face Types (Using Data From the Neutral Examples From the 19 Breeds, *n* = 475)

**Figure 10:**
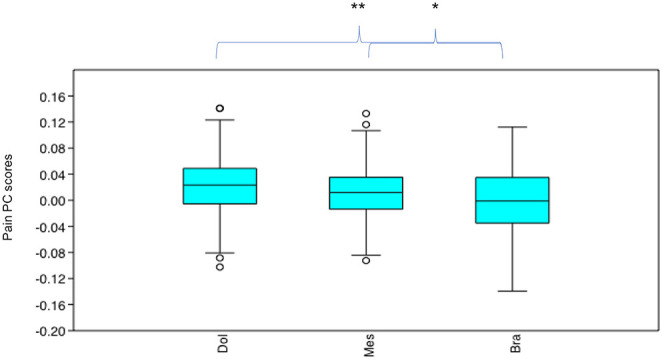
Boxplot with minimum, first quartile, median, third quartile, maximum values and outliers for Pain PC scores for each cephalic type, across 19 breeds (*n* = 475). Dol, dolichocephalic; Bra, brachycephalic; Mes, mesocephalic. *p < 0.05, **p < 0.01. A lower Pain PC is indicative of greater pain-like features present.

Pain PC scores varied significantly across the neutral face examples according to their cephalic type (*x*^2^ = 14.64, *p* = 0.0006612). Pain PC scores were significantly lower (indicating greater presence of pain-like features) in the brachycephalic face examples compared to both mesocephalic (*u* = 1.255E04, *p* = 0.01049) and dolichocephalic (*u* = 8,412, *p* = 0.001958) faces, which were not significantly different to each other (*u* = 9,972, *p* = 0.578), see Figure 10.

### Differences in Pain PC Scores Between T2 (“In pain” DSH Cats), T3 (“No Pain” DSH cats) and the Neutral Examples of Different Breeds (*n* = 525), see Supplementary Data 6 for Full Results of Mann-Whitney *U*-Tests

**Figure 11:**
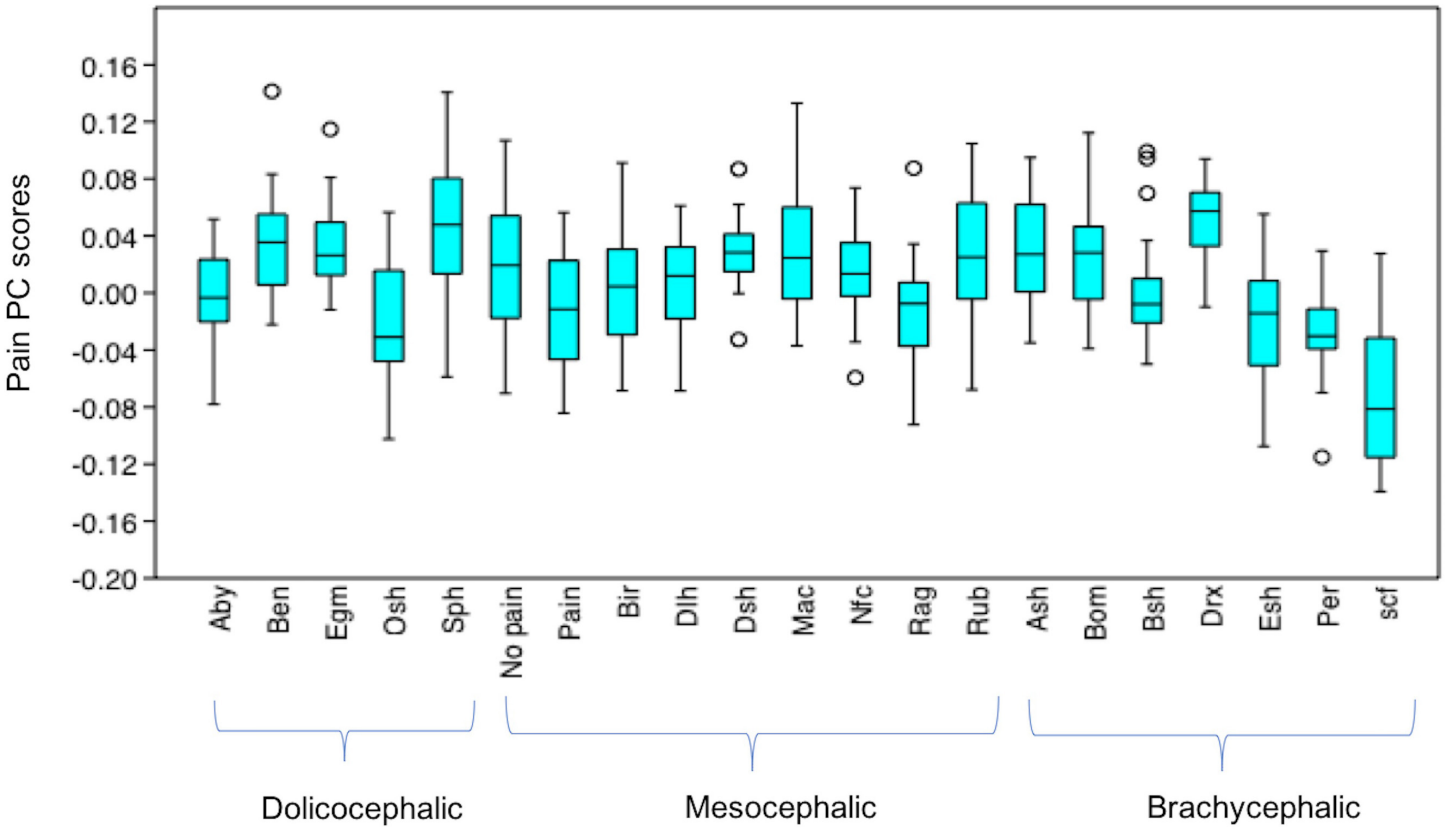
Boxplot with minimum, first quartile, median, third quartile, maximum values and outliers for Pain PC scores for T2 (“pain” DSH population), T3 (“no pain” DSH population) and the 19 different breeds (*n* = 525). A lower Pain PC is indicative of greater pain like features present. Abbreviations for breeds within each cephalic type as follows. Dolichocephalic: Aby, Abyssinian; Ben, Bengal; Egm, Egyptian Mau; Osh, Oriental Shorthair; Sph, Sphynx. Mesocephalic: Bir, Birman; Dlh, Domestic Longhair; Dsh, Domestic shorthair; Mac, Main Coon; Nfc, Norwegian Forest cat; Ragdoll, Russian Blue. Brachycephalic: Ash, American Short hair; Bom, Bombay; Drx, Devon Rex; Esh, Exotic Short hair; Per, Persian; Scf, Scottish fold. For full results of Mann Whitney *U*-tests see Supplementary Data 6. For shape changes associated with higher Pain PC scores, see (45).

After Bonferroni correction, pain PC scores for T2 (“in pain” DSH cats) were significantly different from the neutral examples of the different breeds in only a handful of instances. T2 scores were not significantly different from the scores of any of the mesocephalic breed examples (all *p* > 0.05). T2 scores were significantly different from 29% (two of the seven) brachycephalic breeds, being lower (indicating greater pain-like features) compared to Devon Rex (*u* = 62, *p* = 0.0002583), but higher compared to Scottish Fold scores (*u* = 114, *p* = 0.02565). T2 scores were significantly different to 60% (3 of the 5) dolichocephalic breeds, being lower compared to the Bengal (*u* = 120, *p* = 0.04096), Egyptian Mau (*u* = 113, *p* = 0.0237) and Sphynx (*u* = 93, *p* = 0.004505) breeds (Figure 11).

The T3, (“no pain”) PC scores were only significantly different to those of Scottish folds, who had lower pain PC scores (*u* = 59, *p* = 0.001923).

Other noticeable results included significantly lower pain PC scores (indicating greater pain-like features), for the neutral faces of several of the brachycephalic breeds (e.g., Exotic short hairs, Persians and particularly Scottish folds) compared to the neutral expressions of most other breeds. In the case of Devon Rex faces however, pain PC scores were generally significantly higher (indicating lower presence of pain-like features) compared to others. PC scores for dolichocephalic cats (in particular Sphynx) indicated comparatively less pain-like features, with the exception of greater pain-like features in Oriental short haired cats. Breeds from the mesocephalic group were generally least varied in their pain PC scores, with the exception of Ragdolls, whose scores were significantly lower (indicating greater pain-like features) compared to DSH cats, several dolichocephalic (Bengal, Egyptian mau and Sphynx), and also brachycephalic breeds (American short hair and Devon rex).

## General Discussion

In accordance with our first aim, we identified key variations in baseline facial landmark configurations within a population of common domestic cat breeds and diverse cephalic shapes. Variation in relative landmark positions were evident at both the cephalic as well as breed level, and were identified across all facial regions. These included the ears, eyes, cheeks, mouth and nose, as represented by the three PCs of focus, their associated lollipop graphs, and the wireframes based on average landmark configurations for each cephalic type and breed. The facial landmarks used in this study were, where possible, located specifically in relation to muscle insertion points, and in all cases positioned relative to areas of the face that are expected to vary as a result of changes in facial expression [see (41, 45)]. A “neutral” or “baseline” face exemplar is considered integral to the reliable detection of specific facial movements, as well as the relative apex of action units (41). The substantial and complex nature of variation in landmark configurations identified in “baseline” face shapes across both cephalic types and breeds, highlights a potential issue concerning the visual identification and isolation of facial shape changes associated with the production of specific expressions and their intensity, at a species level. This is particularly relevant, but potentially not limited to, contexts where individuals' faces are viewed statically (i.e., from images) or where the onset and/or off set of movements associated with a specific expression are missed during real time observations.

The findings relevant to our second aim support this interpretation. We demonstrated that whilst facial landmarks were able to differentiate between “pain” and “no pain” facial features in images of domestic short-haired cats, the “pain” DSH cats were not reliably differentiable from the neutral faces of other breeds, even those with similar (mesocephalic) faces. Additionally, relevant facial landmark positions in the average neutral brachycephalic face shape suggested greater pain-like features compared to the average neutral faces of both mesocephalic and dolichocephalic cats. In the case of Scottish folds, their neutral facial landmarks indicated greater pain-like features even compared to the DSH cats that were actually in pain. Conversely, for Devon Rex cats, as well as several breeds classed as dolichocephalic, their neutral landmark positions indicated a greater absence of pain-like features, compared to the neutral landmarks of various other breeds.

The implications of these findings are potentially relevant not only to the domestic cat (50, 51), but other species where standard visual tools (i.e., grimace scales) are used to identify pain via the face. These scales are generally developed and validated using small and/or homogenous populations [e.g., (60–62)], of which many display variation in their facial morphology at a species level [e.g., (63)], including exaggerated features such as paedomorphy [e.g., (8, 18)]. It is therefore likely that the sensitivity, specificity and general utility of these tools may falter when applied at a species level, particularly where tools are used in “real time” and individuals cannot act as their own controls. In such instances, pain may be over represented in certain populations (potentially some brachycephalic breeds) and underrepresented in others (potentially some dolichocephalic breeds). It may be the case that other visual (e.g., body posture) or general behavior cues may act as more reliable indicators than facial expressions in these instances. Further investigation into the limitations of these tools when applied to morphologically diverse populations is required, which may then necessitate the need to create more cephalic type or even breed specific scales.

This study is, as far as can be ascertained, the first to empirically investigate the influence of anthropocentrically mediated selection on the visual quality of features of communicative value, and in relation to a specific internal state. Collectively, results would suggest that in populations with diverse base-line facial morphology, even to a relatively small degree, the communicative content of individuals' faces may be compromised. The implications of this are potentially far reaching, and may be relevant to other domesticated species with human-influenced facial morphology [e.g., (7, 37, 63)]. These issues of “signal disruption” due to artificial selection are also likely to extend beyond the face, affecting other parts of animals' features employed during visual communication (e.g., their limbs, tails, general body size and shape). Indeed, it has been hypothesized that other communicative modalities such as vocalizations and olfaction have become more heavily relied upon due to the unreliable nature of visual signals (19, 64).

Both dogs (65) and humans (66) are potentially sensitive to differences in the emotional valence of dog's facial expressions, and humans are also able to demonstrate this ability in relation to cats (67). However, performance in such tasks has yet to be assessed in populations with diverse facial morphologically, although the current results would suggest this might be affected. Whilst several studies have investigated the impact of breed-based morphological differences on dogs' behavioral signaling, these have focused on the diversity and frequency of signals produced (such as pawing, biting and vocalizations), rather than their visual presentation *per se* [e.g., (20)]. Important future areas of research therefore include; (i) the quantification of differences in the visual appearance of various body regions in morphologically diverse populations (during social interactions and across different contexts); (ii) assessment of these differences in relation to the use of other non-visual communicative modalities; and (iii) investigation of the impact of these variables on the information extracted by human and conspecific observers.

In accordance with the concept of certain facial features being more attractive because they elicit care-giving behavior (33, 34), for brachycephalic cats, our findings imply that this may extend beyond a paedomorphic appearance, to include features associated with pain expression. The “pain PC” which in this cephalic type indicated greater pain-like expressions, appears to share only minimal overlap (i.e., eye landmarks positioned further down the face and closer to the muzzle) with those typically associated with peadomorphy (i.e., low lying eyes). Such pain-linked features could therefore be considered to have district visual qualities which might function in a separate, but similar, care-giving behavior eliciting capacity.

In dogs, owners report greater levels of attachment to breeds that not only have a more paedomorphic appearance, but can also suffer from chronic, debilitating conditions (68). It may be that the general vulnerable appearance of these animals, potentially combined with their greater dependence on their caretaker, provide various emotional benefits to the care provider [see (69)]. More extreme types of phenomenon, such as Munchausen syndrome by proxy, are documented in the human (70) and to a lesser degree animal literature (71). However, broader investigations of the dynamics relating to pet health, appearance, and owner emotional benefits are required.

The ability of companion animals to readily solicit care from humans is obviously advantageous. However, it is possible that permanently vulnerable looking individuals might have a diminished capacity to clearly indicate when care is or is not required, as well as to display other information relevant to their actual state or intentions. Thus, if certain cat breeds are being selected to display “pain-like” features on their faces, these features may serve to solicit unwanted or inadequate attention from their caregivers. More generally, such types of anthropocentric selection might lead to increased anthropomorphic tendencies (69). If, for example, the animal has the appearance of an expression which humans find relatable on some level, even if it is not necessarily reflective of that animals' affective state, it may be used to attribute emotions or characteristics to them. For example, “grumpy cat” a cat made famous by her coverage on social media (72) achieved her moniker due to her perceived “frowning” facial appearance. However, this was likely a result of a combination of her feline dwarfism and paedomorphic features, rather than an expression of her irritability.

It is important to note that certain brachycephalic breeds might experience degrees of chronic pain or discomfort due to various congenital health conditions (17, 55). Scottish folds are particularly susceptible to painful musculoskeletal problems (40), which might offer an alternate explanation for why their neutral face shapes were linked with greatest pain-like features in this study. Whether these breeds have been selected to display facial features associated with pain, whether they are actually in pain, or a combination of both, warrants further investigation.

When analyzed using a geometric morphometric method, differences in cat's expressions when in pain were comparatively subtle at the population level, even within a relatively standardized group (45). It is therefore unsurprising that increased diversity in basal facial morphology would interfere with the strength of feature detection. Indeed, in the current study, significant differences in pain PC scores were only evident between the “pain” (T3) and “no pain” (T2) DSH groups when these were analyzed discretely, prior to multiple comparisons and associated *p*-value adjustments. It is likely that the “interference” caused by increased diversity in facial morphology extends to various other facial shape changes and expressions within their repertoire, particularly those that may be similarly subtle in their presentation, even at an individual level [e.g., see catFACS; (41)]. In the current study, the baseline faces of cats across the different breeds were given a pain score that was previously developed to assess pain-linked expressions in domestic short haired cats (45). Important next steps are therefore to assess whether the faces of other breeds do actually change shape in similar ways to DSH cats when in pain, as well as the degree to which these occur, compared to baseline. Similar investigations should also extended to other affective states [e.g., fear, frustration, relaxed engagement (49)].

It could be argued that as a recently domesticated species from predominantly asocial ancestors (73, 74), the domestic cat may not possess the functional or motivational prerequisites to communicate in relatively subtle or socially complex ways. This could potentially account for some of the lack of differentiation between the faces of DSH cats in pain and the other breeds. Indeed, domestic cats do not possess the facial musculature necessary to produce the eyebrow raising movement that in dogs may convey adoption advantages (31, 38). Furthermore, in contrast to dogs, cat adoption does not appear to be contingent upon their facial expressions, only their overt communicative gestures such as rubbing (42). However, the fact that clear and differentiable facial expressions have previously been linked with different affective states (49) including pain (45, 50, 51) in this species, as well as the morphologically similar Scottish wild cat (75), suggests that their facial expressions do have communicative value. Whilst it is likely that overt pain expression would have been strongly selected against in cat's ancestors, this is less likely to have extended to their facial expressions, because they are not easily detected at a distance, and would confer distinct advantages in the form of care solicitation during the neonatal period.

While the PC structure relating to pain-linked expressions used in study 2 was previously demonstrated stable within the population it was originally developed [see (45)], its robustness across other pain/no pain DSH populations has yet to be assessed. This is also the case for the stability of PCs quantifying variability across breeds. We fully acknowledge that the classification of cats into breed and cephalic types based solely on the visual appearance of their faces from still images is potentially limited [see (76)]. Furthermore, as no clinical examination was performed on the cats in the images that were used as the “neutral” examples, we also cannot say for certain that all these cats were truly free of pain. However, due to the nature of data collection, it was not possible to generate cephalic indices for all images, gain information on the genotypes of individuals, or obtain information on their health status. Instead, we relied on agreement between experienced judges, the descriptors provided by common breed standards, as well as the judgement of a certified catFACS coder in order to gauge facial neutrality. Additionally, the wireframes generated (based on the average landmark positions) for each cephalic and breed type (Figures 5, 9) appeared suitably visually characteristic of their allocated category. We therefore argue that this method of classification was sufficient for the purposes of demonstrating, in principle, the nature and degree of variability in facial landmarks that are known to vary with the production of facial expressions, across cats with diverse facial morphology.

### Summary

Using the domestic cat as our model, we provide evidence suggestive that variations in the baseline morphology caused by anthropocentric selection might disrupt the communicative content of the face. The implications of these findings are potentially relevant to other species where artificial selection has caused their appearance to diverge from their wild type. Further investigation is required to ascertain the extent to which the functionality of underlying facial musculature is affected by morphological variation, the degree to which other features important for visual communication are also affected, and their prevalence across other domesticated species.

## Data Availability Statement

The original contributions presented in the study are included in the article/Supplementary Materials, further inquiries can be directed to the corresponding author/s.

## Ethics Statement

The animal study was reviewed and approved by Institutional Animal Research Ethical Committee of the FMVZ-UNESP-Botucatu under the protocol number of 20/2008 and the delegated authority of Nottingham Trent University, Research Ethics Committee.

## Author Contributions

LF, MF, and DM contributed to the conception and design of the work. LF and SL generated the data. LF performed the analyses, drafted, and revised the manuscript. MF and DM contributed to an initial draft. All authors contributed to the article and approved the submitted version.

## Conflict of Interest

The authors declare that the research was conducted in the absence of any commercial or financial relationships that could be construed as a potential conflict of interest.
